# Hyaluronan Export through Plasma Membranes Depends on Concurrent K^+^ Efflux by K_ir_ Channels

**DOI:** 10.1371/journal.pone.0039096

**Published:** 2012-06-11

**Authors:** Daniel Hagenfeld, Beatrice Borkenhagen, Tobias Schulz, Hermann Schillers, Udo Schumacher, Peter Prehm

**Affiliations:** 1 Münster University Hospital, Institute of Physiological Chemistry and Pathobiochemistry, Münster, Germany; 2 Muenster University Hospital, Institute of Physiology II, Muenster, Germany; 3 Universitätsklinikum Hamburg-Eppendorf, Institut für Anatomie II: Experimentelle Morphologie, Hamburg, Germany; 4 Section of Periodontology, Department of Conservative Dentistry, Clinic for Oral, Dental and Maxillofacial Diseases, University Hospital Heidelberg, Heidelberg, Germany; University of Patras, Greece

## Abstract

Hyaluronan is synthesized within the cytoplasm and exported into the extracellular matrix through the cell membrane of fibroblasts by the MRP5 transporter. In order to meet the law of electroneutrality, a cation is required to neutralize the emerging negative hyaluronan charges. As we previously observed an inhibiting of hyaluronan export by inhibitors of K^+^ channels, hyaluronan export was now analysed by simultaneously measuring membrane potential in the presence of drugs. This was done by both hyaluronan import into inside-out vesicles and by inhibition with antisense siRNA. Hyaluronan export from fibroblast was particularly inhibited by glibenclamide, ropivacain and BaCl_2_ which all belong to ATP-sensitive inwardly-rectifying K_ir_ channel inhibitors. Import of hyaluronan into vesicles was activated by 150 mM KCl and this activation was abolished by ATP. siRNA for the K^+^ channels K_ir_3.4 and K_ir_6.2 inhibited hyaluronan export. Collectively, these results indicated that hyaluronan export depends on concurrent K^+^ efflux.

## Introduction

Hyaluronan is synthesized at the inner side of cell membranes [1], and is exported by the ABC transporters MRP5 of fibroblasts [2] or CFTR of epithelial cells [3]. Hyaluronan molecules which are typically exported have a molecular weight of 4×10^6^ Da and a diameter of about 300 nm in the fully expanded state enabling them to displace other macromolecules from their territory [4]. It can be retained by CD44 receptor on the outside of the cell membrane, where it reduces the membrane potential [5] or regulates the cell volume [6]. The membrane potential is generated by thin layers of positive and negative charges on either side of the cell membrane. To make transport of polyanions possible, the law of electroneutrality must be obeyed, i.e. cations must follow or even drive hyaluronan export from the cytosol into the extracellular matrix. The most likely cation would be K^+^, because it is the only cation that extrudes in larger quantities and is tightly regulated by a variety of K^+^ channels.

Three major classes of K^+^ channels exist which can be expressed simultaneously [7]. (1) Voltage-driven channels which open once the membrane is depolarized. They govern the repolarisation of neurons after an action potential. (2) K_ir_ channels (inwardly rectifying potassium channels) which serve for a low hyperpolarizing K^+^ exit and are activated upon high extracellular K^+^ concentrations as well as during hyperpolarization. The K_ir_ channels are distinguished into different subgroups consisting of the ATP dependent K^+^ channel and the G-protein activated K_ir_ channel. (3) Ca^2+^-activated K^+^ channels which are activated by high intracellular Ca^2+^ concentrations. For all three categories of channels inhibitors are available which can discriminate them. These blockers were used to analyse for their influence on the membrane potential of human fibroblasts and hyaluronan export.

## Materials and Methods

### Materials

Bis-(1,3-dibutylbarbituric acid) (Di-BAC4(3)) was purchased from Invitrogen, Eugene, USA and other chemicals were obtained from Sigma Chemical Co. The serum-free complete Quantum medium 333 for fibroblasts containing growth factors was bought from PAA Laboratories. [^3^H]glucosamine 30 Ci/mmol was delivered from PerkinElmer.

### Cells and cell culture

Primary cultures of human skin fibroblasts from one donor and the human fibrosarcoma cell line HT1080 were grown in Dulbecco's medium supplemented with streptomycin/penicillin (100 units of each/ml) and 10% foetal calf serum or in serum free Quantum medium supplemented with streptomycin/penicillin (100 units of each/ml) and kanamycin (100 units/ml) on 96 well microtiter plates.

### Determination of the membrane potential

Changes in membrane potential responses were assessed with a fluorometric plate reader as described earlier [8] using the bisoxonol dye bis-(1,3-dibutylbarbituric acid) (Di-BAC4(3)), an anionic potentiometric probe which partitions between cellular and extracellular fluids in a membrane potential-dependent manner. Briefly, cells were grown to near confluency in Dulbecco's medium in 96 well microtiter plates. They were rinsed with 100 μl of Quantum medium containing 1 μg/ml DiBAC4(3) and incubated with the same medium containing varying concentrations of the various drugs. Changes in fluorescence were monitored from the bottom of the wells at excitation and emission wavelengths of 488 and 520 nm, respectively. Depolarisation and hyperpolarisation were reflected by a respective increase or decrease in fluorescence. The resting potential was determined using the method of Krasznai et al. [9]. Fluorescence values were converted into membrane potentials using the Nernst equation E_t_  =  E_0_ −61.5×log f_t_/f_0_, where E_0_ is the resting potential, f_t_ the measured fluorescence, and f_0_ the fluorescence of resting cells at 37°C.

### Hyaluronan synthase activity

The hyaluronan synthase activity was determined on a cell membrane fraction [10]. Fibroblasts were grown to confluence and stimulated by addition of fetal calf serum to a final concentration of 15%. After 5 hours of incubation, the cells were washed with cold phosphate buffered saline (PBS), harvested with the aid of a rubber policeman, sedimented at 1500 g for 5 min and suspended in 30 ml of ice-cold PBS. The cells were then transferred into a Parr-cell disruption bomb, exposed to a nitrogen pressure of 900 psi for 15 min and disrupted by nitrogen cavitation [11]. The particulate fraction was obtained by centrifugation at 40000 g for 20 min. The sediment was suspended in 50 mM TRIS-malonate pH 7.0 at a protein concentration of 200 μg/ml and was mixed with an equal volume of the substrate for hyaluronan synthesis that contained 8 μM UDP-[^14^C] GlcA, 166 μM UDP-GlcNac, 4 mM dithiothreitol, 20 mM MgCl_2_ in 50 mM TRIS-malonate pH 7.0 and incubated at 37°C for 4 hours in the presence of increasing concentrations of multidrug resistance inhibitors. Hyaluronan synthesis was stopped by adding a solution of 10% sodium dodecylsulfate (SDS) to a final concentration of 1%. The mixtures were applied to descending paper chromatography that was developed with ethanol/aq. 1 M ammonium acetate pH 5.5 (13∶7) as solvent. After 18 h the radioactivity of [^14^C] hyaluronan at the origin was determined.

### Determination of hyaluronan synthesis

Human skin fibroblasts were grown to confluence on a surface area of 1100 cm^2^. The cells were stimulated further by adding 5% fetal calf serum for 4 hours. They were washed with cold PBS, scrapped off with a rubber policeman, sedimented by centrifugation for 5 min at 1000 g and suspended in a cold solution of 40 ml of 20 mM TRIS-malonate pH 7.0, 250 mM sucrose. Inside-out vesicles were prepared through nitrogen cavitation [12]. Briefly, the suspension was exposed to 1000 psi of nitrogen pressure in a Parr-Cell Disruption Bomb. After pressure release, the suspension was centrifuged at 2000 g for 6 min. The inside-out vesicles were sedimented from the supernatant by centrifugation at 20.000 g for 15 min. The pellet was again suspended in 1.8 ml of 20 mM TRIS-malonate pH 7.0, 250 mM sucrose. Aliquots of 100 µl were transferred to Eppendorf vials and the vesicles were again sedimented by centrifugation at 14.000 rpm for 10 min. The sediments were suspended in 100 µl of substrate solution for hyaluronan synthesis containing 8 µM-UDP-[14C]- GIcA, 166 µM-UDP-GlcNAc, 20 mM-MgCl2, 10 mM dithiothreitol, 100 mM TRIS-malonate pH 7.0, 250 mM sucrose.

The following solutions were further added:

Control: 100 µl of 100 mM TRIS-malonate pH 7.0, 250 mM sucrose.

Low KCl: 100 µl of 100 mM TRIS-malonate pH 7.0, 250 mM sucrose, 10 mM KCl.

High KCl: 100 µl of 100 mM TRIS-malonate pH 7.0, 300 mM KCl.

Low NaCl: 100 µl of 100 mM TRIS-malonate pH 7.0, 250 mM sucrose, 10 mM NaCl

High KCl, low NaCl: 100 µl of 100 mM TRIS-malonate pH 7.0, 300 mM KCl, 10 mM NaCl.

After incubation at 37°C for 3 hours, the enzymatic reaction was stopped by adding of 10 µl of 10% (w/v) SDS, the solutions were applied to Whatman 3 MM paper and then subjected to descending paper chromatography for 16 hours with ethanol/aqueous 1 M-ammonium acetate, pH 5.5 (13∶7 v/v) as solvent. The paper was dried and the radioactivity at the origin was determined. The experiment was performed in triplicates.

### Size determination of vesicles

An aqueous solution of poly-L-lysine (40 µl) (0.01% w/v, Sigma, Deisenhofen, Germany) was applied to the clean side of a glass bottom petri dish. After 10 min the solution was removed and the petri dish rinsed with water. The vesicle suspension (10 µl) was diluted with 2 ml of 10 mM TRIS-malonate pH 7.0, 0.25 m sucrose on the poly-L-lysine coated petri dishes. The vesicles were stained with the membrane dye FM1-43 (Molecular Probes, Eugene/OR, U.S.A.) at a concentration of 5 µg/ml and image acquisition was performed during dye exposure. Samples were imaged using 100x alpha-Plan FLUAR objective NA 1.45 mounted on a Axio Observer Z1 (Carl Zeiss MicroImaging, Göttingen, Germany). Images were acquired with a back illuminated EM-CCD camera (iXon DU-888, Andor Technology PLC, Belfast/Northern Ireland) controlled by Metamorph (Molecular Devices, Sunnyvale/CA, U.S.A.). ImageJ (NHI, Bethesda/MD, U.S.A.) was used for data analysis. The vesicles were further characterized by the Coulter N4Plus Submicron Particles Sizer.

### Inhibition of hyaluronan export by siRNAs K^+^ channels

The following siRNAs were obtained from Ambion Cambridgeshire, UK:

K_ir_3.4 (s7745) AUAGGUAUCAUGGAAGGUGtt

K_ir_6.2a (s7759)AUAGUGACAAGUGCCUUGUaa

K_ir_6.2b (s7760)UGAUGAUCAUGCUCUUGCGga

K_ir_6.2c (s7761)AAAAAUAACCCAGUACAGGtt

Silencer® Select Negative Control (4390843).

The siRNAs were reverse transfected into HT1080. siRNA solutions (7.5 µl, 10 µM) were pipetted into the wells of 6 well plates, followed by 500 µl of a mixture of 60 µl si-Port in 4 ml serum-free Quantum medium. After 10 min at room temperature, 5 ml of trypsinized cells (5×10^5^ cells/ml) were added and the cells were incubated over night at 37°C. The medium was changed to 2.5 ml fresh Quantum medium and the cells were again incubated for 24 hours at 37°C. The supernatant was withdrawn for determination of the hyaluronan concentration as described above.

### Detection of mRNA by rt-PCR

RT-PCR was performed using the Access RT-PCR System (Promega) and Omniscript® RT Kit (Qiagen) and a Mastercycler® ep realplex (Eppendorf) and the following primers:

K_ir_3.4 forward: 5′-GCCCTTTCCTGCCTTTTTTTC-3′

K_ir_3.4 reverse: 5′-TGCTCCCAGGCTCTGCAGT-3′

K_ir_6.2 forward: 5′-TTGGCAACACCGTCAAAGTG-3′

K_ir_6.2 reverse: 5′-GAGGCGAGGGTCAGAGCTT-3′

### Inhibition of hyaluronan export by exogenous hyaluronan

Human skin fibroblasts were grown in 96 well microtiter plates to near confluency. The media were replaced by serum-free Quantum media containing 1 nCi [^3^H] glucosamine and a serial 1∶1 dilution of hyaluronan. After incubation at 37°C for 24 hours, the culture supernatant was subjected to descending paper chromatography for 16 hours with ethanol/aqueous 1 M-ammonium acetate, pH 5.5 (13∶7 v/v) as solvent. The paper was dried and the radioactivity at the origin was determined. The experiment was performed in duplicates. Digestion with hyaluronidase before paper chromatography removed the radioactivity from the origin indicating that it consisted of [^3^H] hyaluronan.

## Results

### Is hyaluronan export linked to membrane potential?

**Figure 1:**
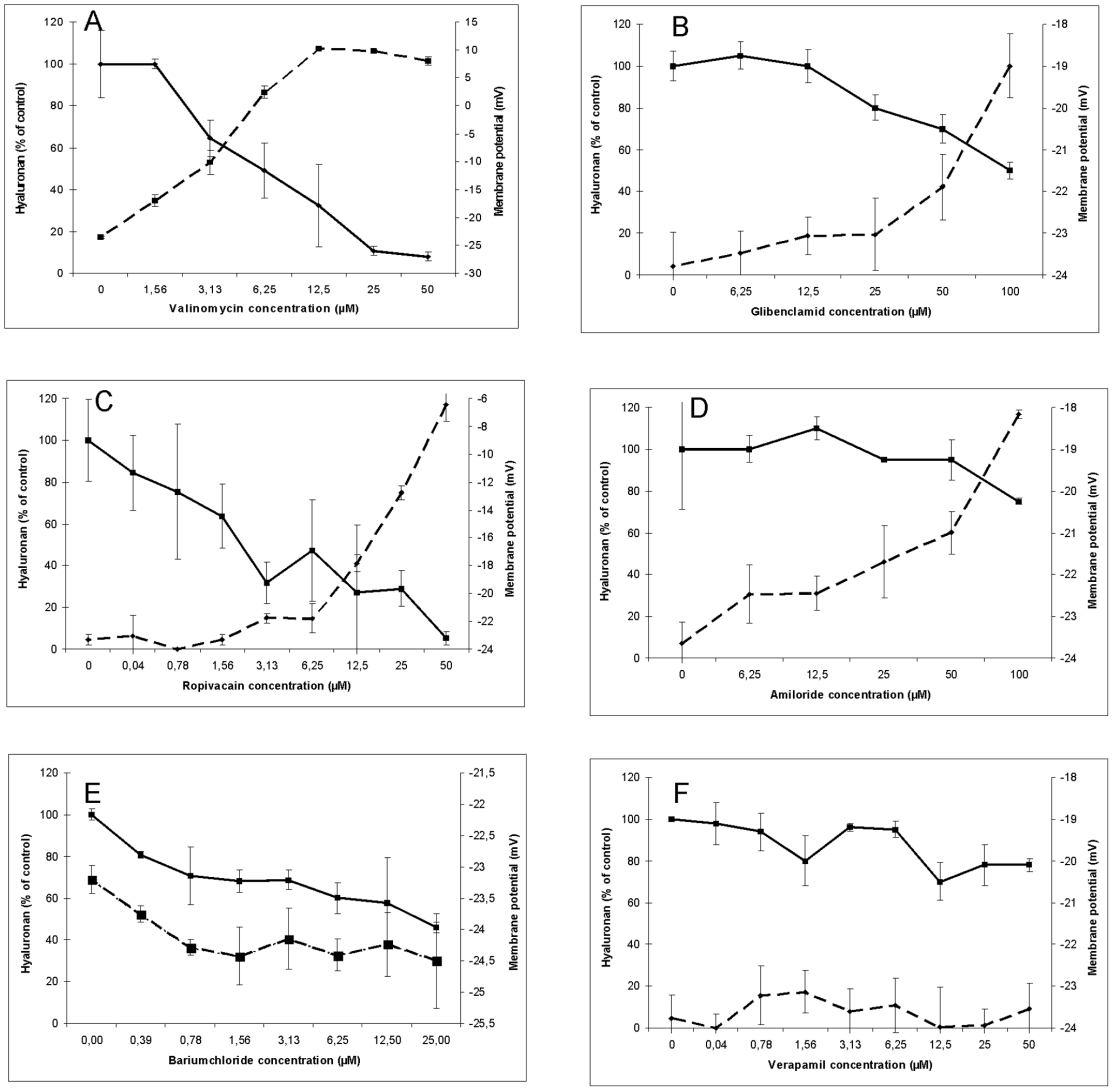
Effect of K^+^ export inhibitors on hyaluronan export and membrane potential. Human skin fibroblasts were grown in 96 well microtiter plates with increasing concentrations of valinomycin (A), glibenclamid (B), ropivacaine (C), amiloride (D), barium chloride (E) and verapamil (F) for 24 hours. The membrane potentials (- -) and hyaluronan concentrations (__) were determined as described in the methods section. The error bars indicate the sd of 3 determinations.

A set of K^+^ blocking drugs was used in a concentration dependent manner to simultaneously analyse the influence on the membrane potential and on hyaluronan export in cultures of human skin fibroblasts. Valinomycin is highly selective for K^+^ and facilitates its movement through the plasma membrane. Fig. 1A shows that valinomycin both reduced hyaluronan export and membrane potential. Glibenclamid inhibits ATP-sensitive K^+^ channels [13]. Fig 1B shows that it reduced the membrane potential in a concentration dependent manner from −24 mM to −19 mV at 100 µM and inhibited hyaluronan export by 50%.

Ropivacaine (Naropin®) is also an inhibitor of ATP-sensitive channels [14]–[16]. Fig. 1C shows that it reduced the membrane potential in a concentration dependent way from −24 mM to −6 mV at 100 µM and also inhibited hyaluronan export by 80%.

Amiloride directly blocks the epithelial sodium channel (ENaC) thereby inhibiting sodium reabsorption and indirectly reducing K^+^ export as well as the membrane potential [17]. Fig. 1D shows that it decreased the membrane potential in a concentration dependent manner from – 24 mM to −18 mV at 100 µM and blocked hyaluronan export by 20%.

Barium chloride is a reversible inhibitor of K_ir_ channels [18]; [19]. Fig. 1E shows that increasing concentrations of BaCl_2_ up to 25 µM hyperpolarized the membrane potential marginally and reduced the hyaluronan export by 50%.

Verapamil is a classical calcium antagonist. It reduces the cellular influx of Ca^2+^ [20] and thus has an indirect effect on the Ca^2+^ activated potassium channel. Fig. 1F shows that it did not affect the membrane potential up to concentrations of 50 µM, but reduced hyaluronan export by 20%. The calcium sensitive K^+^ channels were also inhibited directly by specific inhibitors and activators such as charybdotoxin, chlotrimazol, TRAM-34 and NS309. These drugs did not affect hyaluronan export at their effective concentrations for K+ efflux (data not shown).

The above results are summarized in Table 1. They suggested that the membrane potential is not directly correlated with hyaluronan export, as BaCl_2_ hyperpolarized the cells and reduced hyaluronan export and amiloride depolarized and reduce export only marginally. The best correlation existed between the ATP-sensitive K^+^ channels and hyaluronan export.

**Table 1:** Action profile of ion channel inhibitors, percentage of hyaluronan export and alteration of membrane potential at maximal inhibitory concentrations.

Channel Inhibitor	K_ir_		Na^+^	Ca^2+^	HA-inhibition (%)	Potential difference (mV)
	K_ATP_	K_G_				
BaCl_2_	+	+			50	−2
Glibenclamide	+				50	5
Verapamil				+	20	0
Ropivacain	+	+			80	18
Amiloride			+		20	6

### Opening of K+ channels

**Figure 2:**
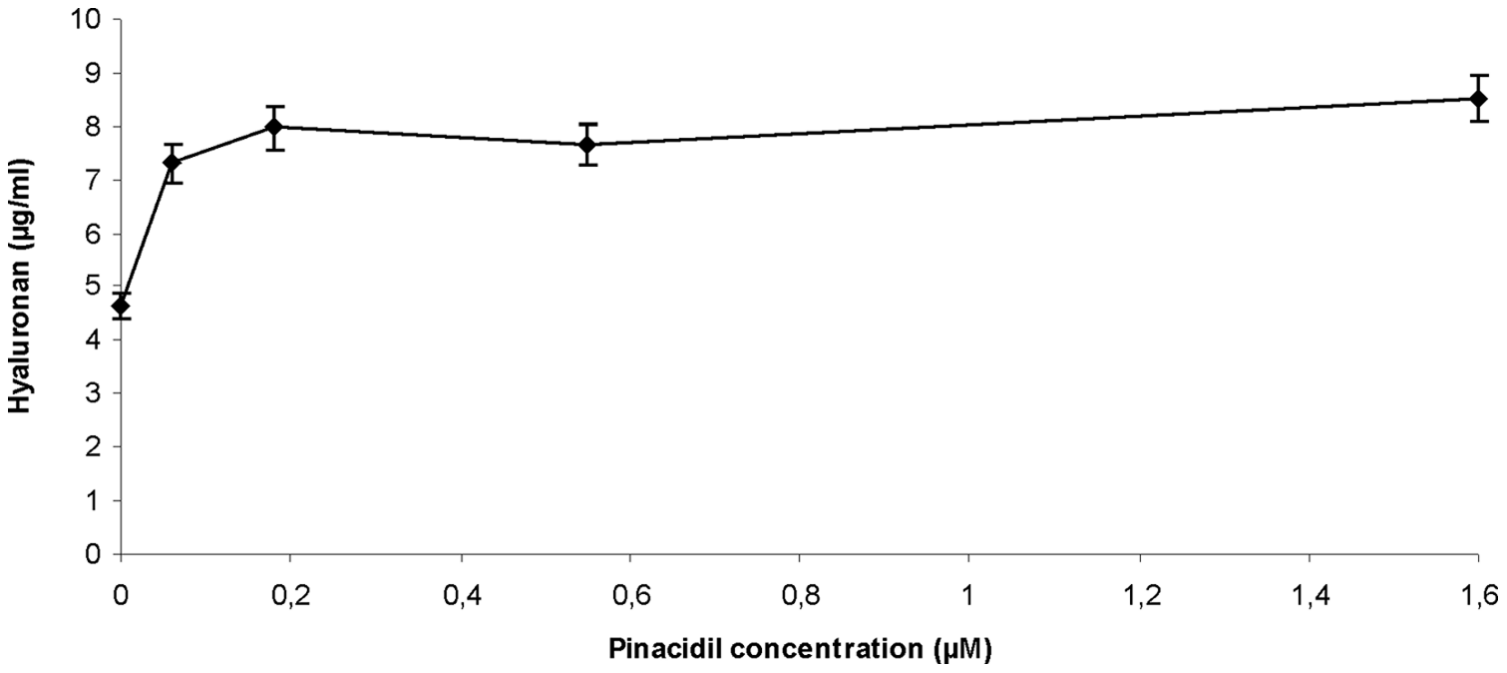
Effect of the K^+^ channel opener pinacidil. Human skin fibroblasts were grown in 96 well microtiter plates with increasing concentrations of pinacidil for 24 hours. The hyaluronan concentrations were determined as described in the methods section. The error bars indicate the sd of 3 determinations.

We investigated in further experiments, whether hyaluronan export may be linked to K^+^ efflux. Pinacidil opens ATP-sensitive K^+^ channels [21]. Fig. 2 shows that it activated hyaluronan export from human skin fibroblasts in a concentration dependent manner verifying that K^+^ channels are indeed involved in hyaluronan export.

### Inhibition by exogenous hyaluronan

**Figure 3:**
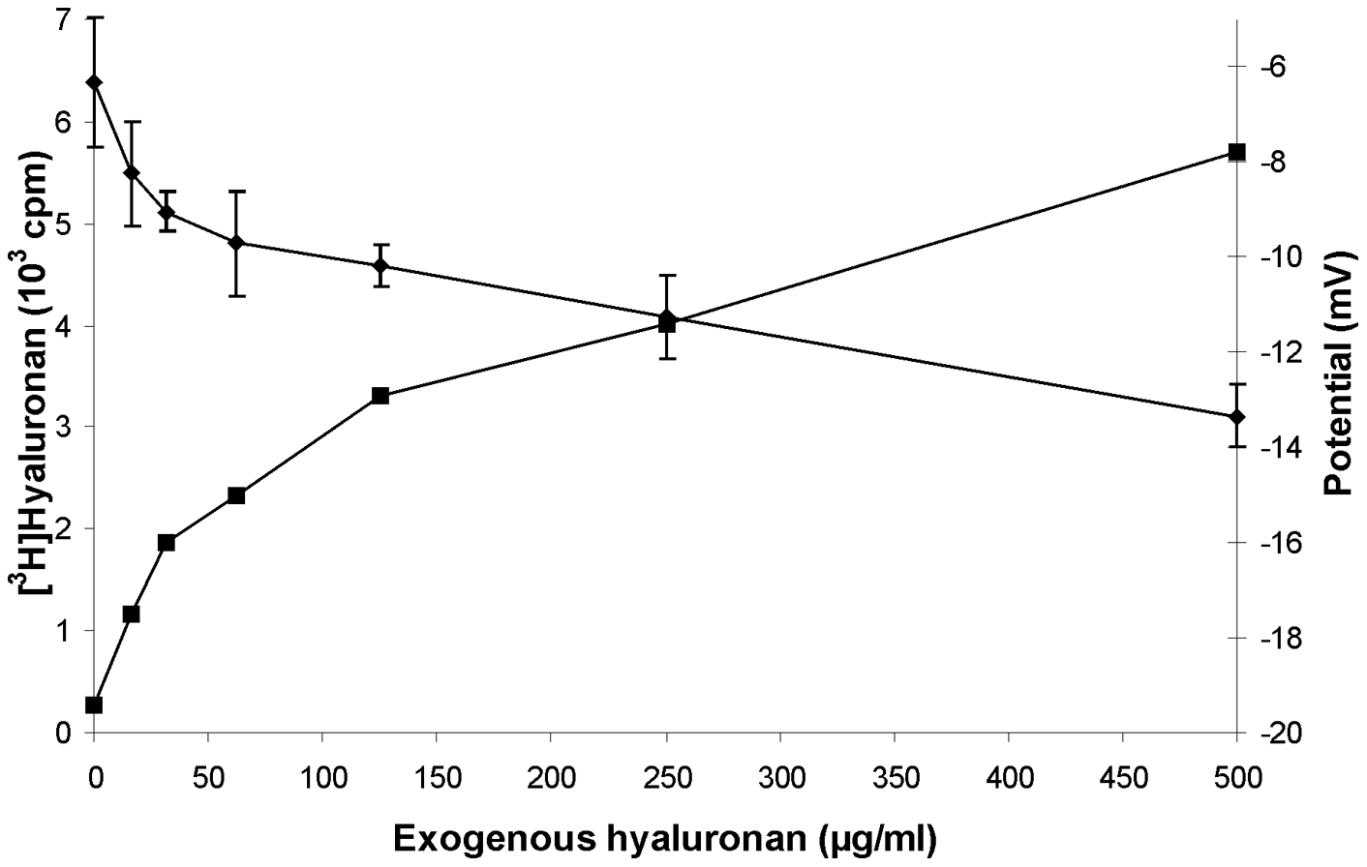
Inhibition by exogenous hyaluronan. Human skin fibroblast were labelled with [^3^H] GlcN and incubated in the presence of the indicated exogenous hyaluronan concentrations. After 24 hours the radioactivity incorporated into [^3^H] hyaluronan was determined. The error bars indicate the mean of duplicate samples.

We recently showed that exogenous hyaluronan reduced the membrane potential and attributed this observation to the Donnan effect of hyaluronan which forces ions back into the cells [5]. Hence, we analysed here, whether exogenous hyaluronan may also affect its own export. Fibroblasts were labelled with [^3^H] glucosamine in the presence of increasing exogenous hyaluronan and shedding of [^3^H] hyaluronan into the culture medium was determined. Fig. 3 shows that exogenous hyaluronan indeed reduced the amount of newly synthesized hyaluronan.

### Is hyaluronan export influenced by K^+^-concentrations on inside-out vesicles?

**Figure 4:**
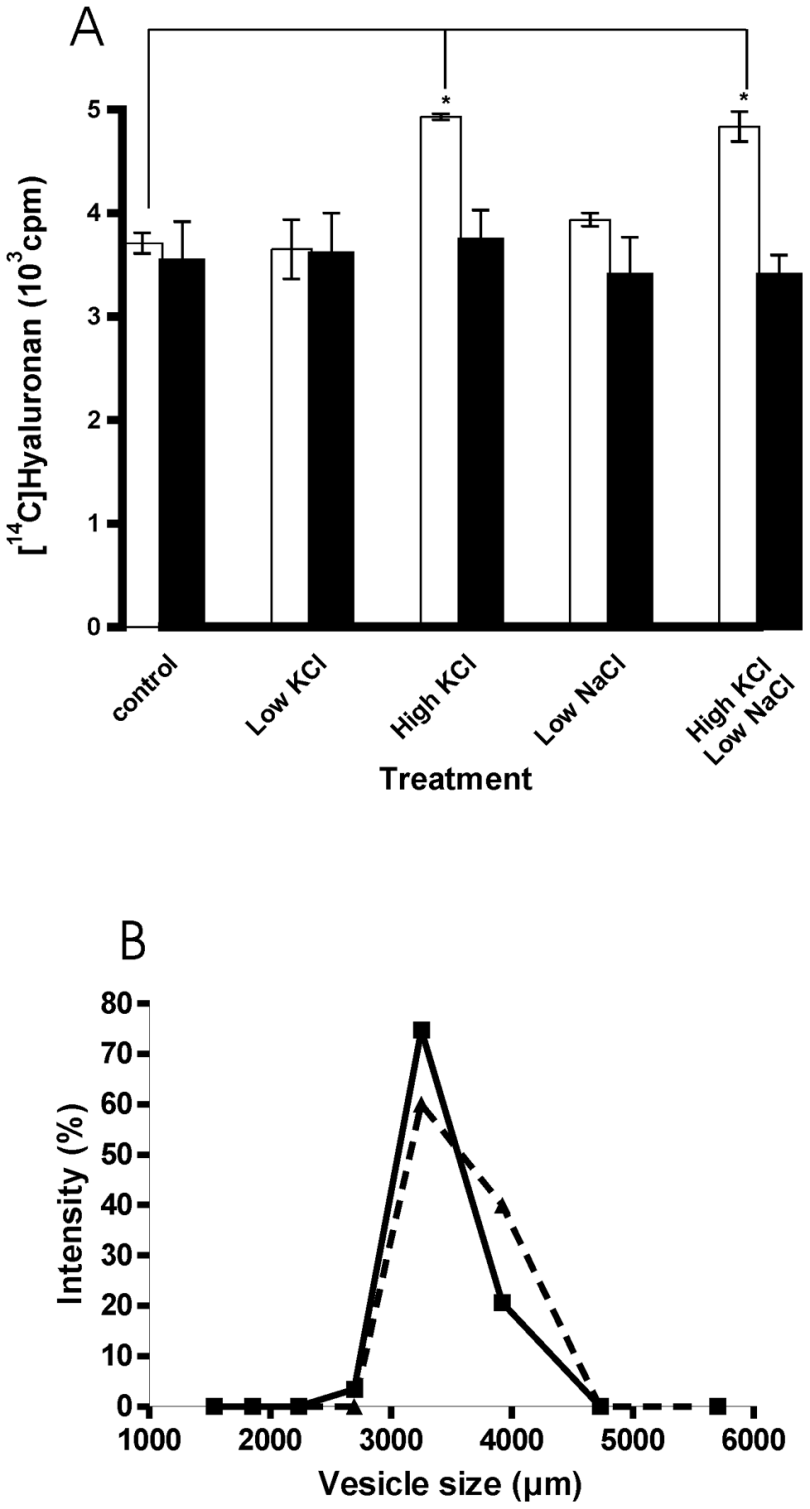
Effect of KCl on vesicular hyaluronan synthesis. Inside-out vesicles were prepared from human skin fibroblasts and incubated for 3 hours with radioactive substrate for hyaluronan synthesis with the salts indicated in the absence (open bars) and presence 10 mM ATP (solid bars). **A.** Radioactivity incorporated into [^14^C] hyaluronan was determined from triplicate samples. *p<0.01 (ANOVA test). **B.** Size determination of vesicles incubated for 2 hours in the absence (- - -) and presence (___) of substrates for hyaluronan synthesis.

The experiments described above could either be explained by complicated cellular signalling pathways or by simple concurrent efflux. In order to discriminate between these alternative hypotheses, inside-out vesicles were prepared from human skin fibroblasts, incubated with the precursor nucleotide sugars UDP-GlcNac and UDP-[^14^C] GlcA in the absence and presence of low (5 mM) and high (150 mM) KCl concentrations and the formation of [^14^C] hyaluronan was determined. Fig. 4A shows that low NaCl or KCl concentrations did not activate hyaluronan synthesis. Only high KCl concentrations were activating. Further activation was not achieved by a mixture of high KCl and low NaCl concentrations which mimic intracellular ion distribution. In another set of experiments, ATP was added at a concentration of 10 mM. This treatment annihilated the activation of hyaluronan import into vesicles by high KCl concentrations. The vesicles were characterized by staining and microscopy. They were closed and heterogeneous. The size distribution was measured by nephelometry using the Coulter N4Plus Submicron Particles Sizer. Fig. 4B shows a comparison of the intensity result. From these data the diameter of the mean intensity peaks were calculated to be 3519 nm for vesicles without hyaluronan synthesis and 3370 nm with hyaluronan synthesis. Thus intravesicular hyaluronan reduced the vesicles size about 4%.

### Inhibition of K^+^ channels by siRNA

**Figure 5:**
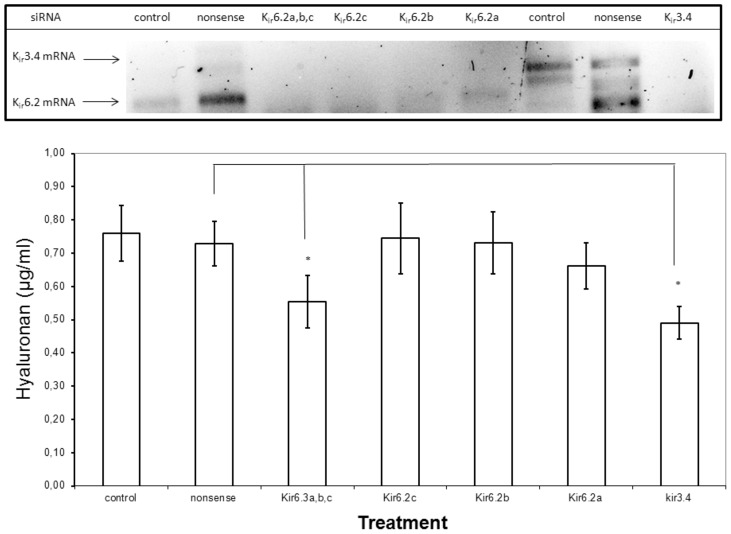
Inhibition of K_ir_ channels by siRNA. Human fibrosarcoma cells HT1080 were reverse transfected with 30 nM siRNA for K_ir_3.4, three different K_ir_6.2 siRNAs, a mixture of the three K_ir_6.2 siRNAs or non-sense siRNA and incubated for 24 hours in serum free Quantum medium. **A.** Specific inhibition of mRNA. The downregulation of K_ir_3.4 mRNA and K_ir_6.2 mRNA was detected by rtPCR. **B.** The hyaluronan concentrations were determined in the culture media. The error bars indicate the standard error of 6 determinations. *p<0.01 (ANOVA test).

Human fibroblasts express the G-protein gated K_ir_3.4 and the ATP-sensitive K_ir_6.2 channels [22]–[24]. Therefore we chose these channels to analyse their knock-down on hyaluronan export in the HT1080 cell line, because these cells proved to be more suitable for cellular transfection. Fig. 5 shows that K_ir_3.4 siRNA and a mixture of three K_ir_6.2 siRNAs reduced hyaluronan export significantly. The knockdown of the respective mRNAs was controlled by rtPCR.

## Discussion

The transport of charged molecules through plasma membranes requires the stoichiometric cotransport of counterions in order to fulfill the law of electroneutrality, and this applies particularly to macromolecules such as hyaluronan. Nature has designed several mechanisms for cotransport. Secondary-active transporter proteins mediate uphill transport of a solute by tapping into the free energy provided by the concentration gradient of a coupled ion that is specific to the transporter protein (e.g., H^+^, K^+^, or Na+). Chloride transport by CFTR is believed to be linked to Na^+^ transport by the epithelial sodium channel (ENaC). If cotransport is stoichiometrically coupled, there is a driving force of the membrane potential in addition to downhill diffusion along the concentration gradient.In our previous studies, we recognized that hyaluronan export was sensitive to inhibitors of K^+^ channels [10]. Since K^+^ channels are also responsible for membrane potential formation, we investigated the relationship in closer detail. A set of different K^+^ channel inhibitors revealed that the membrane potential was not correlated with hyaluronan export in all cases. Prominent inhibition of hyaluronan export over 50% was observed with glibenclamide, ropivacain and barium chloride which are all rather specific for K_ir_ channels.

The inhibition of hyaluronan production by exogenous hyaluronan could also rely on K^+^ flow through plasma membranes. We have previously shown that exogenous hyaluronan reduced the membrane potential, caused blebbing and volume increase by its Donnan effect that forced salt into the cells thereby reversing the normal efflux of K^+^ [5]; [6]. The influx of ions did not only affect K^+^ ions but also Ca^2+^, as has recently been shown for postsynaptic L-type Ca^2+^ channels [25]. On the other hand, treatment of fibroblasts with hyaluronidase caused hyperpolarisation, volume decrease and stimulation of hyaluronan production [5]; [6]; [26].

To eliminate the possibility that inhibition was caused by intracellular signalling cascades or the toxicity of drugs, hyaluronan synthesis and transport were analysed in inside-out vesicles. Import of hyaluronan into these vesicles was activated only by high KCl (150 mM) and this activation was thwarted by the addition of ATP. This result indicated that hyaluronan import was dependent on ATP-sensitive K^+^ channels which are blocked by increased intracellular ATP levels. The activation by KCl occurred on a high basic background as compared to the control which could be due to faster hyaluronan synthesis than transport leading to excessive extravesicular loops. It is also possible that other K_ir_ channels participated in K^+^ efflux. In control experiments the vesicles were examined microscopically and appeared to be closed. The size distribution as determined by nephelometry was about 3.5 µm in diameter and decreased about 4% upon incubation with substrates for hyaluronan synthesis. Thus intravesicular hyaluronan decreased the vesicle size. We observed a similar phenomenon on intact cells incubated with inhibitors of hyaluronan export [6].

The participation of K^+^ channels in hyaluronan export was verified using siRNA of the G-protein gated K_ir_3.4 and the ATP-sensitive K_ir_6.2 channels. This could arise from the properties of both channels to form functional heterodimers [27].

Our results show that there is an uncoupling of membrane potential and hyaluronan export. A similar observation for the interactions of Kv1.3 channels and cell surface integrins was made previously. This was correlated to the K^+^ efflux rather than the modulation of the membrane potential per se [28]. An explanation for this dissociation could be the microheterogeneity on the cell membrane. It is known that hyaluronan synthesis is confined to membrane protrusions [29], whereas the membrane potential is measured on the whole cell. There is an interesting correlation of clustering of K^+^ channels [30] and hyaluronan [31] in neurons. The glucose induced formation of ATP is known to depolarize membranes of microvilli, but not basolateral membranes in the small intestine [32]. High glucose concentrations can also reduce the amount of hyaluronan produced by gingival fibroblasts [33] and epidermal keratinocytes [34].

Our results suggest that cells have an additional level of regulating hyaluronan production by the K^+^ efflux through K^+^ channels besides transcriptional regulation of the synthase or the MRP5 transporter namely their covalent modification and allosteric inhibition. Regulation by ATP-sensitive K^+^ channels may operate to control the level of intracellular hyaluronan during entrance of dividing cells into mitosis, where high levels of intracellular ATP [35] as well as hyaluronan were found [36]; [37]. In addition, elevated hyaluronan synthesis as well as enhanced expression of several K^+^ channels are correlated in some metastatic tumour cells [38]; [39]. Our results propose a mechanistic explanation for these observations. The metabolic association of hyaluronan synthesis and K^+^ conductivity may also have great impact on neurotransmission.

**Figure 6:**
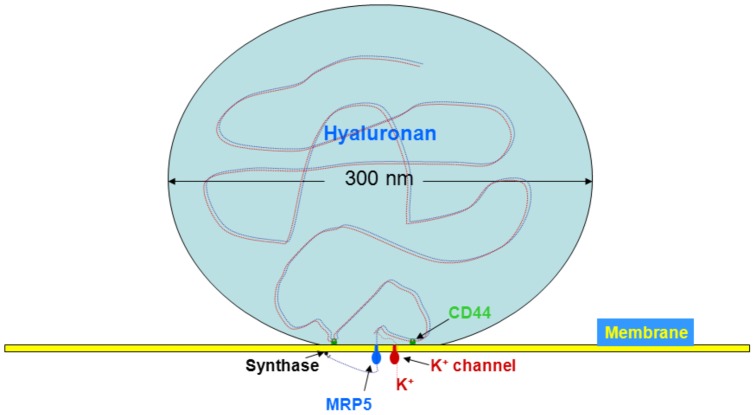
Model of hyaluronan synthesis and export. The hyaluronan synthase assembles hyaluronan at the inner side of the plasma membrane, the chains are exported by the ABC transporter MRP5 from fibroblasts and retained on the cell surface by CD44. Concurrent K^+^ efflux is required for maintaining electroneutrality.

It is interesting to consider the topographic dimensions of hyaluronan on the MRP5 export site of plasma membranes (Fig. 6). Hyaluronan molecules typically exported have a molecular weight of 4×10^6^ Da and a diameter of about 300 nm in the fully expanded state that readily displaces other macromolecules from its territory. The chain is retained by CD44 receptor on the plasma membrane with a thickness of about 7 nm. The membrane potential is generated by thin layers of positive and negative charges on either side of the membrane. Hyaluronan is thread through a pear like MRP5 structure with breadths of 6 nm to 13 nm, a length of about 13 nm and a hole of 2.5 nm [40]; [41]. Thus nascent hyaluronan chain occupies a large area of the membrane which can be regarded as a closed system in the physico-chemical sense. If a charged molecule is transported through a membrane in a closed system, the law of electroneutrality has to be obeyed; e. g. concurrent cationic counter ion export is required. This requirement is fulfilled by the K^+^ channels located in the vicinity of the hyaluronan exporter.

## References

[1] Prehm P (1984). Hyaluronate is synthesized at plasma membranes. Biochem.J..

[2] Schulz T, Schumacher U, Prehm P (2007). Hyaluronan export by the ABC-transporter MRP5 and its modulation by intracellular cGMP. J Biol.Chem..

[3] Schulz T, Schumacher U, Prante C, Sextro W, Prehm P (2010). Cystic Fibrosis Transmembrane Conductance Regulator Can Export Hyaluronan. Pathobiology..

[4] Laurent TC (1964). The interaction between polysaccharides and other macromolecules. The exclusion of molecules from hyaluronic acid gels and solutions.. Biochem.J.

[5] Hagenfeld D, Schulz T, Ehling P, Budde T, Schumacher U (2010). Depolarisation of the membrane potential by hyaluronan. J.Cell Biochem..

[6] Joerges J, Schulz T, Wegner J, Schumacher U, Prehm P (2012). Regulation of cell volume by glycosaminoglycans. J.Cell Biochem..

[7] Estacion M (1991). Characterization of ion channels seen in subconfluent human dermal fibroblasts.. J.Physiol.

[8] Gopalakrishnan M, Miller TR, Buckner SA, Milicic I, Molinari EJ (2003). Pharmacological characterization of a 1,4-dihydropyridine analogue, 9-(3,4-dichlorophenyl)-3,3,6,6-tetramethyl-3,4,6,7,9,10-hexahydro-1,8(2H,5 H)-acridinedione (A-184209) as a novelK(ATP) channel inhibitor. Br.J Pharmacol..

[9] Krasznai Z, Marian T, Balkay L, Emri M, Tron L (1995). Flow cytometric determination of absolute membrane potential of cells.. J Photochem.Photobiol.B.

[10] Prehm P, Schumacher U (2004). Inhibition of hyaluronan export from human fibroblasts by inhibitors of multidrug resistance transporters. Biochem.Pharmacol..

[11] Klempner MS, Mikkelsen RB, Corfman DH, Andre SJ (1980). Neutrophil plasma membranes. I. High-yield purification of human neutrophil plasma membrane vesicles by nitrogen cavitation and differential centrifugation. J.Cell Biol..

[12] Schlemmer SR, Sirotnak FM (1995). Facile preparation of inside-out plasma membrane vesicles from tumor cells for functional studies of pharmacologically relevant translocating ATPases. Anal.Biochem..

[13] Ashcroft FM (2005). ATP-sensitive potassium channelopathies: focus on insulin secretion.. J.Clin.Invest.

[14] Dojo M, Kinoshita H, Nakahata K, Kimoto Y, Hatano Y (2004). Effects of bupivacaine enantiomers and ropivacaine on vasorelaxation mediated by adenosine triphosphate-sensitive K(+) channels in the rat aorta.. Anesthesiology.

[15] Kawano T, Oshita S, Takahashi A, Tsutsumi Y, Tomiyama Y (2004). Molecular mechanisms of the inhibitory effects of bupivacaine, levobupivacaine, and ropivacaine on sarcolemmal adenosine triphosphate-sensitive potassium channels in the cardiovascular system.. Anesthesiology.

[16] Friederich P, Solth A (2004). Interaction of ropivacaine with cloned cardiac Kv4.3/KChIP2.2 complexes.. Anesthesiology.

[17] Kleyman TR, Cragoe EJ (1988). The mechanism of action of amiloride. Semin.Nephrol..

[18] French RJ, Shoukimas JJ (1985). An ion's view of the potassium channel. The structure of the permeation pathway as sensed by a variety of blocking ions.. J.Gen.Physiol.

[19] Franchini L, Levi G, Visentin S (2004). Inwardly rectifying K+ channels influence Ca2+ entry due to nucleotide receptor activation in microglia.. Cell Calcium.

[20] Baky SH, Singh BN (1982). Verapamil hydrochloride: pharmacological properties and role in cardiovascular therapeutics.. Pharmacotherapy.

[21] Katsuda Y, Egashira K, Ueno H, Arai Y, Akatsuka Y (1996). ATP-sensitive K+ channel opener pinacidil augments beta 1-adrenoceptor-induced coronary vasodilation in dogs.. Am.J.Physiol.

[22] Li GR, Sun HY, Chen JB, Zhou Y, Tse HF (2009). Characterization of multiple ion channels in cultured human fibroblasts. PLoS.ONE..

[23] Chilton L, Ohya S, Freed D, George E, Drobic V (2005). K+ currents regulate the resting membrane potential, proliferation, and contractile responses in ventricular fibroblasts and myofibroblasts.. Am.J Physiol Heart Circ.Physiol.

[24] Davison HR, Taylor S, Drake C, Phuan PW, Derichs N (2011). Functional Fluorescently Labeled Bithiazole DeltaF508-CFTR Corrector Imaged in Whole Body Slices in Mice. Bioconjug.Chem..

[25] Kochlamazashvili G, Henneberger C, Bukalo O, Dvoretskova E, Senkov O (2010). The Extracellular Matrix Molecule Hyaluronic Acid Regulates Hippocampal Synaptic Plasticity by Modulating Postsynaptic L-Type Ca(2+) Channels. Neuron..

[26] Philipson LH, Westley J, Schwartz NB (1985). Effect of hyaluronidase treatment of intact cells on hyaluronate synthetase activity.. Biochemistry.

[27] Ishihara K, Yamamoto T, Kubo Y (2009). Heteromeric assembly of inward rectifier channel subunit Kir2.1 with Kir3.1 and with Kir3.4. Biochem.Biophys.Res.Commun..

[28] Levite M, Cahalon L, Peretz A, Hershkoviz R, Sobko A (2000). Extracellular K(+) and opening of voltage-gated potassium channels activate T cell integrin function: physical and functional association between Kv1.3 channels and beta1 integrins. J.Exp.Med..

[29] Rilla K, Tiihonen R, Kultti A, Tammi M, Tammi R (2008). Pericellular Hyaluronan Coat Visualized in Live Cells With a Fluorescent Probe is Scaffolded by Plasma Membrane Protrusions. J Histochem.Cytochem..

[30] Antonucci DE, Lim ST, Vassanelli S, Trimmer JS (2001). Dynamic localization and clustering of dendritic Kv2.1 voltage-dependent potassium channels in developing hippocampal neurons.. Neuroscience.

[31] Meszar Z, Felszeghy S, Veress G, Matesz K, Szekely G (2008). Hyaluronan accumulates around differentiating neurons in spinal cord of chicken embryos. Brain Res.Bull..

[32] Luppa D, Hartenstein H, Muller F (1987). Relation between microvilli membrane potential and glucose transport capacity of rat small intestine.. Biomed.Biochim.Acta.

[33] Willershausen-Zonnchen B, Lemmen C, Hamm G (1991). Influence of high glucose concentrations on glycosaminoglycan and collagen synthesis in cultured human gingival fibroblasts. J.Clin.Periodontol..

[34] Jokela TA, Lindgren A, Rilla K, Maytin E, Hascall VC (2008). Induction of hyaluronan cables and monocyte adherence in epidermal keratinocytes. Connect Tissue Res..

[35] Marcussen M, Larsen PJ (1996). Cell cycle-dependent regulation of cellular ATP concentration, and depolymerization of the interphase microtubular network induced by elevated cellular ATP concentration in whole fibroblasts.. Cell Motil.Cytoskeleton.

[36] Brecht M, Mayer U, Schlosser E, Prehm P (1986). Increased hyaluronate synthesis is required for fibroblast detachment and mitosis. Biochem.J..

[37] Evanko SP, Parks WT, Wight TN (2004). Intracellular Hyaluronan in Arterial Smooth Muscle Cells: Association with Microtubules, RHAMM, and the Mitotic Spindle. J Histochem.Cytochem..

[38] Toole BP (2004). Hyaluronan: from extracellular glue to pericellular cue.. Nat.Rev.Cancer.

[39] Arcangeli A, Crociani O, Lastraioli E, Masi A, Pillozzi S (2009). Targeting ion channels in cancer: a novel frontier in antineoplastic therapy. Curr.Med.Chem..

[40] Ravna AW, Sylte I, Sager G (2008). A molecular model of a putative substrate releasing conformation of multidrug resistance protein 5 (MRP5). Eur.J Med.Chem..

[41] Ravna AW, Sylte I, Sager G (2007). Molecular model of the outward facing state of the human P-glycoprotein (ABCB1), and comparison to a model of the human MRP5 (ABCC5). Theor.Biol.Med.Model..

